# Novel Golden Lipid Nanoparticles with Small Interference Ribonucleic Acid for Substrate Reduction Therapy in Fabry Disease

**DOI:** 10.3390/pharmaceutics15071936

**Published:** 2023-07-12

**Authors:** Marina Beraza-Millor, Julen Rodríguez-Castejón, Jonatan Miranda, Ana del Pozo-Rodríguez, Alicia Rodríguez-Gascón, María Ángeles Solinís

**Affiliations:** 1 Pharmacokinetic, Nanotechnology and Gene Therapy Group (Pharma Nano Gene), Faculty of Pharmacy, Centro de Investigación Lascaray Ikergunea, University of Basque Country UPV/EHU, 01006 Vitoria-Gasteiz, Spain; marina.beraza@ehu.eus (M.B.-M.); julen.rodriguez@ehu.eus (J.R.-C.); ana.delpozo@ehu.eus (A.d.P.-R.); 2 Bioaraba, Microbiology, Infectious Disease, Antimicrobial Agents and Gene Therapy, 01006 Vitoria-Gasteiz, Spain; 3 GLUTEN3S Research Group, Faculty of Pharmacy, University of Basque Country UPV/EHU, 01006 Vitoria-Gasteiz, Spain; jonatan.miranda@ehu.eus; 4 Bioaraba, Nutrition and Food Safety, 01006 Vitoria-Gasteiz, Spain

**Keywords:** substrate reduction therapy, solid lipid nanoparticles, siRNA, Fabry disease, Gb3 synthase, gold nanoparticles

## Abstract

Substrate reduction therapy (SRT) has been proposed as a new gene therapy for Fabry disease (FD) to prevent the formation of globotriaosylceramide (Gb3). Nanomedicines containing different siRNA targeted to Gb3 synthase (Gb3S) were designed. Formulation factors, such as the composition, solid lipid nanoparticles (SLNs) preparation method and the incorporation of different ligands, such as gold nanoparticles (GNs), protamine (P) and polysaccharides, were evaluated. The new siRNA–golden LNPs were efficiently internalized in an FD cell model (IMFE-1), with GNs detected in the cytoplasm and in the nucleus. Silencing efficacy (measured by RT-qPCR) depended on the final composition and method of preparation, with silencing rates up to 90% (expressed as the reduction in Gb3S-mRNA). GNs conferred a higher system efficacy and stability without compromising cell viability and hemocompatibility. Immunocytochemistry assays confirmed Gb3S silencing for at least 15 days with the most effective formulations. Overall, these results highlight the potential of the new siRNA–golden LNP system as a promising nanomedicine to address FD by specific SRT.

## 1. Introduction

Fabry disease (FD) is a monogenic X-linked lysosomal storage disorder (LSD) caused by mutations in GLA gene [1], which codifies for the lysosomal enzyme α-galactosidase A (α-Gal A). As a result, the enzymatic activity decreases, and there is a progressive accumulation of the glycosphingolipid globotriaosylceramide (Gb3) and its deacylated derivate globotriaosylsphingosine (lysoGb3) within lysosomes of multiple cell types. FD is a multisystemic disease, mainly affecting the kidneys, heart and nervous system [2]. The current treatments for FD include enzyme replacement therapy (ERT) and an oral pharmacological chaperone. ERT, which consists of the intravenous administration of the recombinant enzyme every other week, represents the first specific therapeutic approach in patients with FD. The two ERT commercialized products, agalsidase alfa (Replagal^®^) and agalsidase beta (Fabrazyme^®^), show limited efficacy due to low tissue penetration, not crossing the blood–brain barrier, infusion-associated reactions, as well as the formation of neutralizing antidrug antibodies in a high proportion of patients [3,4]. The chaperone migalastat (Galafold^®^), approved in 2016 by the European Medicines Agency (EMA), binds to the active site of α-Gal A and improves functionality of the patient’s own enzyme. However, migalastat is only effective in amenable mutations, i.e., missense mutations with a preserved residual enzyme function [5,6]. Other strategies for FD are currently under research, such as second generation ERT (pegunigalsidase-alfa and moss-derived α-Gal A), substrate reduction therapy (SRT), stem cell gene mRNA therapy and gene supplementation [3,7]. 

**Figure 1:**
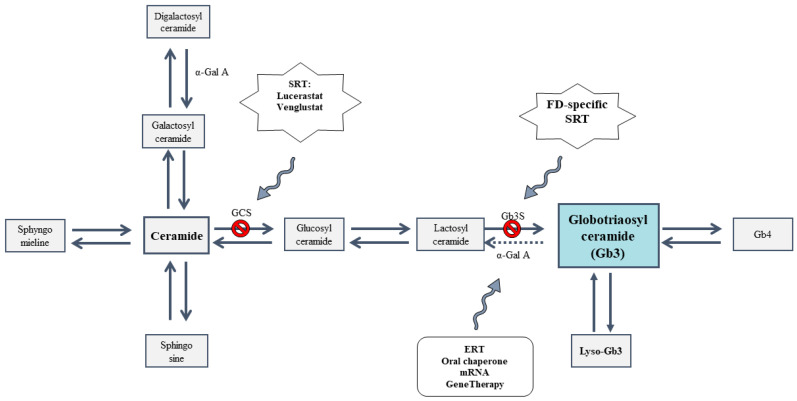
Application of substrate reduction therapy (SRT) and enzyme replacement therapy (ERT) on the metabolic pathway of glycosphingolipids. GCS: Glucosylceramide synthase. α-Gal A: α-Galactosidase A. Gb3S: Gb3 Synthase. FD: Fabry disease. Gb3: Globotriaosylceramide. Gb4: Globotetraosylceramide.

SRT has been proposed to reduce the glycosphingolipids accumulation in LSDs. For FD, SRT may be used to prevent the de novo formation of Gb3 [8]. Currently, there are two SRT products under clinical research: the iminosugar analogue lucerastat (NCT03425539, NCT03737214) and venglustat (NCT02228460, NCT02489344). Both act as orally available inhibitors of glucosylceramide synthase (GCS), preventing the accumulation of Gb3 by limiting the amount of ceramide (Figure 1) [9,10]. 

Inhibition of Gb3 formation at the level of GCS has been related to side effects because it also leads to suppression of other glycosphingolipids, such as glucosylceramide and lactosylceramide [11]. An alternative to treat FD by SRT may be the suppression of Gb3 synthesis in a later step; in this sense, reduction in the enzyme Gb3 synthase (Gb3S) has been proposed as a FD-specific SRT.

Recently, silencing therapies based on interference RNA (iRNA) have emerged as tools for SRT. Small interference RNAs (siRNAs) are short double-stranded RNAs composed of 21–23 base pairs with two overhanging phosphorylated bases at the 3′ end of each strand. siRNA molecules are instable, and sufficiently small (7–8 nm in length and 2–3 nm in diameter) to undergo renal clearance while being too large (~13 kD) for membrane passage and cellular uptake [12]. Therefore, the success of siRNA therapies depends on the development of delivery systems able to protect the nucleic acid and to facilitate its interaction with the target cell. Lipid nanoparticles (LNPs) have been proposed as tunable carrier systems for delivering nucleic acids, including siRNA. Many studies have found LNPs to be effective, generally safe, and well-tolerated. LNPs also present advantages in terms of production since they can be manufactured by easy and scalable methods. Patisiran, the first siRNA therapy approved [13], and the COVID-19 vaccines are good examples of LNP-based products recently approved by the Food and Drug Administration (FDA) and by the EMA. Among LNPs, cationic liposomes are the most studied carriers for siRNA delivery [12,14]. Apart from liposomes, solid lipid nanoparticles (SLNs), which are spherical particles with a solid lipid core composed of well-tolerated physiological lipids, are also a promising delivery system for siRNA. In fact, the capacity of SLNs for nucleic acid delivery, including pDNA, mRNA and shRNA, has been previously demonstrated [15,16,17,18,19]. Among siRNA delivery vehicles, gold nanoparticles (GNs) have also been extensively studied due to their advantages in terms of biocompatibility, manufacturing, high surface-to-volume ratio, and simplicity of surface functionalization [14,20]. 

In this work, we propose the development of a novel nanomedicine for the treatment of FD by specific SRT, based on the administration of siRNAs targeted to Gb3S. With this aim in mind, siRNA nanodelivery vectors based on SLNs and GNs (golden LNPs) were designed. Formulation factors such as the composition, SLNs preparation method and the addition of different ligands were optimized. After an extensive physicochemical characterization of the formulations, the intracellular behavior of these novel golden LNPs and their efficacy to silence the enzyme were evaluated in a FD cell model.

## 2. Materials and Methods

### 2.1. Materials 

1-Dioleoyl-3-trimethylammonium-propane chloride salt (DOTAP) and 1,2-dioleoyl-3-dimethylammonium-propane (DODAP) were obtained from Avanti Polar-lipids, Inc. (Alabaster, AL, USA). Gattefossé (Madrid, Spain) kindly provided Precirol^®^ ATO 5. Tween 80 and dichloromethane were purchased from Panreac (Madrid, Spain). Protamine sulfate salt from salmon (Grade X) (P), dextran (Mw of 3.26 kDa) (DX) and D-Galacto-*D*-mannan from Ceratonia siliqua (Mr~200,000) were provided from Sigma-Aldrich (Madrid, Spain), as well as Nile Red. 5 nm diameter GNs were obtained from Sigma-Aldrich (Madrid, Spain) and 1.8 nm diameter gold nanoparticles were purchased from Creative Diagnostics (New York City, NY, USA).

siRNAs against Gb3S mRNA were synthesized at a PerkinElmer company (formerly Dharmacon^TM^, Cambridge, UK). Three functional siRNAs with the following sequences were tested (from 5′ to3′): GGACACGGACUUCAUUGUU (J-016315-05) (siRNA 1), GCACUCAUGUGGAAGUUCG (J-016315-06) (siRNA 2) and UGAAAGGGCUUCCGGGUGG (J-016315-08) (siRNA 3). A scrambled sequence (UGGUUUACAUGUCGACUAA) was used as negative control. Dharma^®^FECT transfection reagent was also obtained from Dharmacon^TM^ (Cambridge, UK). 

For the agarose gel electrophoresis, materials were obtained from Bio-Rad (Madrid, Spain). Agarose was purchased from Sigma-Aldrich (Madrid, Spain), GelRed^TM^ from Biotium (Fremont, CA, USA) and GeneRuler Ultra Low Range DNA Ladder from Gibco (Thermo Fisher Scientific, Waltham, MA, USA). 

IMFE-1 cells (immortalized Fabry endothelial cell line-1) were employed for cell culture studies. Cell culture medium was obtained from Lonza (Basel, Switzerland), including EGM^®^-2 supplemented with 2% fetal bovine serum (FBS), hydrocortisone, human epidermal growth factor (hEGF), vascular endothelial growth factor (VEGF), human fibrobastic growth factor (hFGF-B), insulin-like growth factor 1 (R3-IGF-1), gentamicin sulfate-amphotericin (GA-1000), ascorbic acid and heparin. Other cell culture reagents, including trypsin/EDTA and phosphate-buffered saline (PBS) were purchased from Gibco (Thermo Fisher Scientific, Madrid, Spain). 

To quantify gene expression, a quantitative reverse transcription PCR (RT-qPCR) was performed using the LightCycler^®^ 2.0 System (Roche Diagnostics, Mannheim, Germany). The materials employed for the RT-qPCR were also provided by Roche Diagnostics (Mannheim, Germany). 

For the viability assay, thiazolyl blue tetrazolium bromide (MTT) was obtained from Sigma-Aldrich (Madrid, Spain) and isopropyl alcohol was purchased from Panreac (Madrid, Spain).

For the immunocytochemistry assay, Anti-Gb3S rabbit antibodies were provided by Sigma-Aldrich (Madrid, Spain), as well as Triton^TM^ X-100 and goat serum. Goat–anti-rabbit IgG cross-adsorbed secondary antibody (Alexa Fluor^TM^ 488) was purchased from Gibco (Thermo Fisher Scientific, Madrid, Spain). 4′, 6′-diamine-2′phenylindole dihydrochloride (DAPI)-fluoromount g was obtained from Southern Biotech (Birmingham, AL, USA). In addition, a Label IT^®^ Cy^®^5 Nucleic Acid Labeling Kit was obtained from Mirus (Madison, WI, USA).

### 2.2. Preparation of SLNs and Vectors

SLNs were prepared by two different techniques previously reported: hot-melt emulsification (SLN_HM_) [21] and solvent evaporation/emulsification (SLN_EE_) [22]. SLN_HM_ were prepared by mixing an oily phase of Precirol^®^ ATO 5 and an aqueous solution containing Tween 80, and either DOTAP or a mixture of DOTAP and DODAP, both heated at 80 °C for 30 min. For SLN_EE_, Precirol^®^ ATO5 was dissolved in dichloromethane (5% *w/v*), mixed with the aqueous solution and sonicated for 30 s, then the dichloromethane was evaporated.

siRNA vectors were prepared with GNs, P, either Dx or Gal, and SLNs, as shown in Figure S1. The order of addition as well as the proportions of each component were previously optimized. Although other combinations with the same components were prepared, we selected only those vectors that proceeded to be stable (in terms of size and surface charge) for at least one week at 4 °C (Table S1). Vectors prepared with 20 nm GNs were ruled out due to their high particle size (≈600 nm) and neutral or slightly negative surface charge.

### 2.3. Size, Polidispersity Index and ζ-Potential Measurement

Size and polydispersity index (PDI) were measured by dynamic light scattering (DLS), and ζ-potential by laser doppler velocimetry (LDV), in a Zetasizer Nano ZS (Malver Instruments, Worcestershire, UK). 

### 2.4. Stability Study of siRNA-Based Vectors: Size, PDI and ζ-Potential

Vectors were stored at 4 °C for 30 days. Then, particle size, PDI and ζ-potential were measured. 

### 2.5. Morphology Studies of siRNA Vectors

#### 2.5.1. Transmission Electron Microscopy (TEM) Images

A Philips EM208S TEM with an Olympus SIS purple digital camera (Olympus Life Science, Tokyo, Japan) was used for negative staining transmission electron microscopy (TEM). Ten µL of the sample were placed on glow discharged carbon coated grids, and allowed to settle for 60 s before being blotted away by filter paper. Staining was carried out using 2% uranyl acetate for 60 s. 

#### 2.5.2. Cryo-Transmission Electron Microscopy (Cryo-TEM) Images

Cryo-transmission electron microscopy (Cryo-TEM) was performed using a TECNAI G2 20 TWIN (FEI), operating at an accelerating voltage of 200 KeV in a bright-field image mode and low-dose image mode. Three µL of the sample was placed on glow-discharged 300 mesh Quantifoil TEM grids and used for plunge freezing into liquid ethane on an FEI Vitrobot Mark IV (Eindhoven, The Netherlands). The frozen grids were transferred to a 626 DH Single Tilt Cryo-Holder (Gatan, France), where the temperature was below −170 °C, and then to TEM at liquid nitrogen temperature.

### 2.6. Agarose Gel Electrophoresis Assay

Agarose gel electrophoresis was performed in an agarose gel (2%) labelled with Gel Red^TM^ with an Uvitec Uvidoc D-55-LCD-20 M Auto transilluminator (Uvitec, Cambridge, UK). The vectors presenting 0.02 µg siRNA/µL were electrophoresed at 75 V for 50 min. RiboRuler Ultra low Range DNA Ladder was used as a control.

### 2.7. In Vitro Studies in IMFE-1 Cells

In vitro studies were carried out in a cell model of FD (IMFE-1 cells) [23]. This cell line presents the R112H mutation in α-Gal A gene, which supposes an α-Gal A activity significantly lower than that of other non-Fabry cells (16% of activity). The cells were kindly provided by Dr. Christine R. Kaneski (National Institute of Neurological Disorders and Stroke National Institutes of Health, Bethesda, MD, USA) and Dr. Shen (Institute of Metabolic Disease, Baylor Research Institute, Dallas, TX, USA). For transfection studies, vectors were diluted in HBS, and 75 µL were added to cultured cells. Complete medium was refreshed 4 h post-transfection.

#### 2.7.1. Cellular Uptake

For entry assay, SLNs were labelled with Nile Red. Nile Red altered neither the particle size nor the zeta potential of the SLNs. Vectors were added to the cultured cells in a total siRNA concentration of 50 nM. Two hours after transfection, cells were washed twice with PBS and detached from plates. The cellular uptake was analyzed using a CytoFLEX flow cytometer (Beckman Coulter, Brea, CA, USA) at 610 nm. 

To evaluate the intracellular location of the Nile-Red-labelled vectors, cells were seeded and incubated for 24 h in 4-well Millicell EZ slides (Millipore). Two hours after the addition of the vectors, the slides were washed twice with PBS, fixed with PFA 4% and covered with the mounting fluid DAPI-fluoromount-G^®^. 

#### 2.7.2. Intracellular Disposition of the siRNA and GNs

Label^®^IT Cy^®^5-labelled siRNA vectors were prepared to study the intracellular disposition of siRNA. Cells were treated with a final siRNA concentration of 100 nM. Two hours after transfection, the samples were fixed similarly to cellular uptake study. 

The intracellular disposition of GNs was evaluated by TEM. IMFE-1 cells were treated with the vectors at a final siRNA concentration of 50 nM. Twenty minutes after transfection, cells were prefixed with 0.5% (*v/v*) glutaraldehyde, detached and centrifuged at 800× *g* for 5 min to obtain a compact pellet, and 2% (*v/v*) glutaraldehyde was added. The fixed pellet was washed three times with 0.1 M Sorensen buffer and 4–8% (*w/v*) sucrose, and postfixed with 1% OsO_4_ in phosphate buffer for 2 h at 4 °C in darkness. The sample was dehydrated following increasing series with acetone and epoxy-acetone. Before ultramicrotomy (1 µm), the sample was introduced in Beem capsules and polymerized with new epoxy resin.

#### 2.7.3. Cell Viability

MTT Analysis was performed for viability analysis. Metabolic activity was measured using absorbance analysis by a GloMax^®^-Multi + Detection System (Promega, Madison, WI, USA). 

#### 2.7.4. Silencing Efficacy

The capacity of the vectors to silence Gb3S was evaluated in IMFE-1 cells- Different siRNA concentrations (15–50 nM) in the culture medium were tested. In preliminary studies, by using DharmaFECT^®^ and a SLN-based vector, siRNA 1 reduced the Gb3s mRNA expression, but to a lesser extent than siRNA 2 and 3 (Figure S2). Therefore, we started the optimization of our vectors with the less effective siRNA (siRNA1). For silencing studies, vectors were added for a final siRNA concentration of 25 nM. 

Gb3S mRNA expression in cells was measured as previously described by Zumbrun et al. [24]. Forty-eight hours post-transfection, total RNA was extracted from cells using a High Pure RNA Isolation Kit with DNAse digestion and reverse-transcribed using the Transcriptor First Strand cDNA Synthesis Kit. The cDNA was amplified using the LightCycler^®^ FastStart DNA Master SYBR Green I with specific primers sets to quantify the human Gb3S transgene and a primer set specific to the β-actin gene as the endogenous reference. To quantify transgene expression for human Gb3S 5′-GGCAACATCTTCTTCCTGGAGACTTC-3′ and 5′-CGAACTTCCACATGAGTGCGATCC-3′ were used as specific sense and antisense primers, respectively. The primer set specific for the β-actin gene (5′-CATTGTGATGGACTCCGGAGACGG-3′, sense; 5′-CATCTCCTGCTCGAAGTCTAGAGC-3′, antisense) served as an endogenous reference. The thermal cycling conditions were 95 °C for 600 s, followed by 45 cycles of 95 °C for 5 s, 62 °C for 10 s and 72 °C for 20 s. At the end, melting curve was performed by a single cycle of 95 °C for 60 s, 70 °C for 60 s and 95 °C for 1 s. Quantification of mRNA levels, and thus the silencing inhibition potential, was calculated using the ∆∆Ct method. As negative control of Gb3S gene silencing efficacy, ON-TARGETplus^TM^ non-targeting siRNA was employed (scramble). ON-TARGETplus^TM^ Cyclophilin B Control siRNA (Human) was used to confirm the silencing effect.

#### 2.7.5. Gb3S Enzyme Expression

Immunocytochemistry assay was performed to detect Gb3S in IMFE-1 cells. Vectors were added in a final siRNA concentration of 25 nM. After 7 or 15 days of incubation, cells were washed twice with PBS and fixed with PFA 4%. They were incubated with a primary rabbit antibody specific for Gb3S (Sigma-Aldrich, Madrid, Spain) at 4 °C. Twelve hours after, the goat-anti-rabbit IgG secondary antibody was added for one hour. Finally, they were covered with the mounting fluid DAPI-fluoromount-G^®^.

### 2.8. Interaction with Erythrocytes: Hemolysis and Hemagglutination

Hemolytic and hemagglutination effect of the vectors were assessed following the protocol described by Kurosaki et al. [25]. Fresh blood was centrifuged at 4000 rpm during 5 min, and the plasma and the buffy coat were discarded. Erythrocytes were washed three times with PBS by centrifugation. Finally, they were diluted in PBS to a final concentration of 5% (*v/v*) for the hemolysis study and of 2% (*v/v*) for agglutination evaluation. Vectors were mixed with the erythrocyte suspension at a ratio 1:1 (*v/v*) and incubated 60 min for hemolysis analysis. After centrifugation at 4000 rpm for 5 min, hemoglobin released in the supernatant was measured at 545 nm using a Glomax^®^-Multi Detection System (Promega, Madison, WI, USA) microplate reader. Hemolysis values were expressed as percent respect to the lysis buffer positive control (100%).

For agglutination study, vectors were incubated with the erythrocyte suspension at a ratio of 1:1 (*v/v*) for 15 min. Then, 15 µL of the mixture were placed on a microscope slide. A vector prepared with poly-L-lysine was used as positive control. Samples were observed by an optic inverted microscope (Nikon TS, Izasa Scientific, Madrid, Spain).

### 2.9. Data Analysis and Statics

Results are expressed as the mean ± standard deviation. All statistical computations were performed using IBM^®^ SPSS^®^ Statics 28 (IBM Corp, Armonk, NY, USA). The normal distribution of samples was assessed by the Shapiro–Wilk test, and homogeneity of variance by the Levene test. The different formulations were compared with ANOVA and Student’s *t*-test. Difference were considered statistically significant at *p* < 0.05.

## 3. Results and Discussion

### 3.1. Size, PDI and ζ-Potential of the SLNs and Vectors

SLNs are regarded as one of the most effective nonviral vectors for nucleic acid delivery [26,27] with advantages such as biocompatibility and the ease of large-scale production, among others [15]. Two different techniques have been used to prepare SLNs, EE and HM, with the avoidance of organic solvents being one important advantage of the HM. A solid lipid core of Precirol ATO5^®^ was present in all formulations. In some of them, the lipid core was surrounded by the cationic lipid DOTAP (nanoparticle stabilizer), whereas in others, the ionizable lipid DODAP was also included. Table 1 shows the composition, particle size, PDI and ζ-potential of the four types of SLNs prepared. Significant differences (*p* < 0.001) in size and zeta potential were observed among all formulations. Not only the preparation method, but also the composition affected the physicochemical properties of SLNs, which are key factors for efficacy. The longer and high-energy homogenization process may be responsible of the smaller particle size of SLN_HM_ [28]. For the same preparation method, the inclusion of DODAP resulted in a higher particle size, probably due to the lesser surfactant capacity of the DODAP respect to DOTAP. All SLNs showed a positive ζ-potential favoring the electrostatic interactions with the negatively charged siRNA. PDI values were lower than 0.3 in all cases, which indicates homogeneity in particle size. 

**Figure 2:**
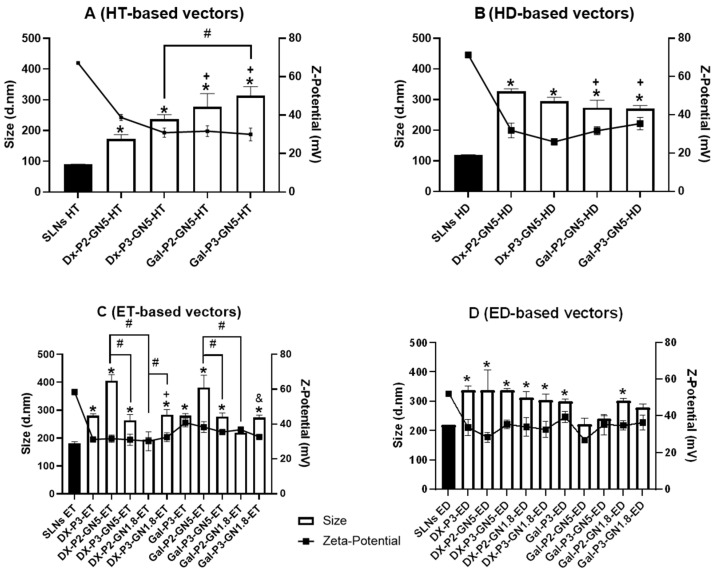
Size and zeta-potential of the siRNA vectors. Data are expressed as mean ± standard deviation; *n* = 3. (**A**): HT-based vectors. * *p* < 0.001 with respect to SLN_HT_. + *p* < 0.001 with respect to Dx-P2-GN5-HT. # *p* < 0.001 with respect to the other formulation. (**B**): HD-based vectors. * *p* < 0.001 with respect to SLN_HD_. + *p* < 0.001 with respect to Dx-P2-GN5-HD. (**C**): ET-based vectors. * *p* < 0.001 with respect to SLN_ET_. + *p* < 0.001 with respect to Dx-P2-GN5-ET. & *p* < 0.001 with respect to Gal-P2-GN5-ET. # *p* < 0.001 with respect to the other formulation. (**D**): ED-based vectors. * *p* < 0.001 with respect to SLN_ED_.

SLN_HM_ and SLN_EE_, with the same composition, showed differences in the size, ζ-potential and surface to mass ratio. Those features condition the electrostatic interaction and the auto-assembly with the other components of the final vector. Figure 2 and Table S2 show the size, PDI and ζ-potential of the SLN-based siRNA vectors. This is the first time that a vector containing GN and SLNs has been employed for siRNA delivery. The ability to form stable SLN-based vectors depended on the method of preparation of SLNs. Figure 2 and Table S2 show the size, PDI and ζ-potential of the SLN-based siRNA vectors. The ability to form stable SLN-based vectors depended on the method of preparation of SLNs. When SLN_HM_ were used, the presence of GNs was necessary to form stable siRNA vectors. On the contrary, it was possible to form vectors without GNs by increasing the proportion of P in the final system, although the inclusion of GNs improved their physicochemical characteristics. In fact, the presence of P was necessary to reduce the final size of all the nanoformulations (<500 nm). The size range of the vectors prepared with DOTAP + DODAP (221–337 nm) (Figure 2B,D) was smaller than those prepared with DOTAP (173–406 nm) (Figure 2A,C). 

The final vectors also contained a polysaccharide, Dx or Gal, that confers stability, stealth properties, and helps to modulate the cell internalization [29,30]. The type of polysaccharide influenced the particle size of the vectors prepared with SLN_HM_ (Figure 2A,B), whereas the proportion of P highly influenced the particle size of the nanoformulations prepared with SLN_ET_ (Figure 2C). In the case of Dx-P2-GN5-ET/Dx-P3-GN5-ET and Gal-P2-GN5-ET/Gal-P3-GN5-ET, the lower P ratio resulted in the larger particle size (*p* < 0.001). On the contrary, for Dx-P2-GN1.8-ET/Dx-P3-GN1.8-ET, the higher the P ratio, the larger the particle size (*p* < 0.001). Surface charge was always positive (≈+35 mV) and lower than that of the precursor SLNs as a result of the adsorption of the siRNA-(GN)-P-Dx or Gal complexes on the surface of the SLNs. 

The addition of GNs to the nanovectors prepared by EE technique did not lead to a change of the particle size respect to the same vector without GNs. However, when the golden LNPs were prepared with the lower proportion of P and SLN_ET_, differences between those containing GNs of 5 or 1.8 nm were observed (*p* < 0.001). 

PDI values of the vectors were higher than those of the precursor SLNs, ranging between 0.31 and 0.45. A decrease in zeta potential was observed for all vectors compared to SLNs (*p* < 0.001), although the final surface charge was always positive, from +28.5 mV to +41 mV.

### 3.2. Stability Study of siRNA-Based Vectors: Size, PDI and ζ-Potential

In general, all siRNA vectors stored at 4 °C for 30 days presented good stability in terms of size and surface charge (Figure S3). However, in some cases, increases of particle size higher than 50 nm were observed (Dx-P2-GN5-HT, Dx-P3-GN5-HT, Gal-P2-GN5-HD, Gal-P3-GN5-HD, Dx-P2-GN5-ET and Gal-P2-G5-ED). Regarding ζ-Potential, it was quite stable, although in the case of Dx-P2-GN1.8-ET and Dx-P2-GN5-ET, after 30 days, it decreased to about +15 mV and +10 mV, respectively.

### 3.3. Morphology Studies of siRNA Vectors

**Figure 3:**
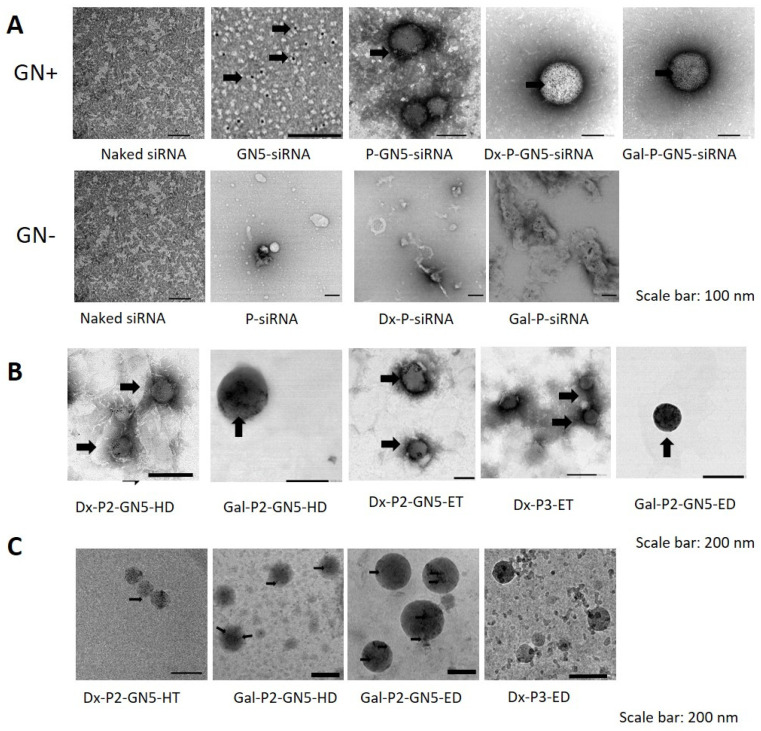
Morphology studies of siRNA vectors. (**A**): TEM images of siRNA naked or combined with the different components of the vectors. (**B**): TEM images of siRNA-based vectors. (**C**): Cryo-TEM images of the siRNA-based vectors. GN: Gold nanoparticles. Black arrows: 5 nm GNs.

Figure 3A shows TEM photographs of the naked siRNA or combined with the different components of the final nanovectors before the addition of SLNs. In GN5-siRNA, black arrows indicate the GNs, which appear as easily detectable black spots. GNs induced the condensation of the siRNA, despite both present negative charge. The chemically active surface of small GNs allows for the conjugation of molecules such as siRNA [31,32]. In addition, GNs can physically interact with the adenine bases present in siRNA, producing a condensation effect [31]. The combination of siRNA, GNs and P (P-GN5-siRNA) led to the formation of spherical particles with the GNs located inside (black arrows). When the polysaccharide (Dx or Gal) was added (Dx-P-GN5-siRNA and Gal-P-GN5-siRNA, respectively), GNs appeared more uniformly distributed, and an electron-dense corona surrounding the structure was observed. In absence of GNs, P was also able to condense the siRNA (P-siRNA). The addition of Dx or Gal led to the formation of structures that were very different from those formed when GNs were also present in the system. 

Figure 3B shows the TEM images of some of the final nanovectors. All of them presented a spherical shape. Black arrows in the image indicate the GNs, that appeared as easily detectable black spots. 

Figure 3C features Cryo-TEM images of some of the vectors. The spherical shape of the vectors with GNs located inside was confirmed when present. The size of vectors from the analysis of the Cryo-TEM photographs was 120 nm for Dx-P2-GN5-HT, 150 nm for Gal-P2-GN5-HD, 240 nm for Gal-P2-GN5-ED and 140 nm for the vector without GNs, Gal-P3-ED. Differences in the size of the vectors were detected depending on the measurement technique. DLS provided higher sizes than TEM and cryo-TEM. The drying process of the samples for TEM imaging may affect the structural integrity of the nanoparticles, particularly, with complex samples [33]. Moreover, negative staining, used to increase contrast for lipid-based nanocarriers, could also produce artifacts in the image [34,35]. On the contrary, cryo-TEM allows for the preservation of the shape and internal structure of particles, but some limitations (possible ice contamination, bias toward small particles due to the fact that it requires an ultrathin film of sample), make Cryo-TEM not amenable as a quantitative method for nanocarrier particle size distribution analysis [36,37,38]. Due to the differences in the measurement principles of each method, a combination of different techniques is convenient [33].

### 3.4. Agarose Gel Electrophoresis Assay

**Figure 4:**
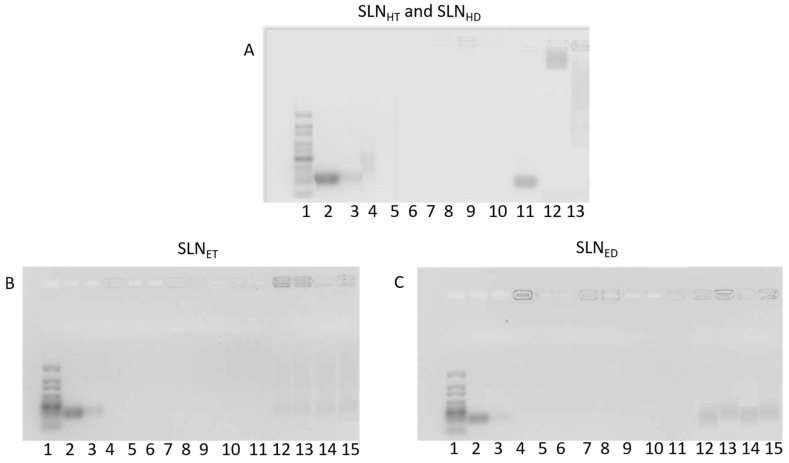
Analysis by agarose gel electrophoresis of the capacity of siRNA-based vectors to bind the siRNA. (**A**): Analysis by agarose gel electrophoresis of the capacity of siRNA-based vectors with hot-melt SLNs to bind the siRNA. (1) 10–300 bp DNA ladder. (2) Naked siRNA. (3) siRNA-GN5. (4) siRNA-GN5-P3. (5) siRNA-GN5-P3-Dx. (6) siRNA-GN5-P3-Gal. (7) Dx-P2-GN5-HT. (8) Gal-P2-GN5-HT. (9) Dx-P2-GN5-HD. (10) Gal-P2-GN5-HD. (11) Dx-P3-HT. (12) Dx-P3GN1.8-HT. (13) DharmaFECT^®^-siRNA. (**B**): Analysis by agarose gel electrophoresis of the capacity of siRNA-based vectors with SLN_ET_ to bind the siRNA. (1) 10–300 bp DNA ladder. (2) Naked siRNA. (3) siRNA- GN5. (4) Dx-P3-ET. (5) Dx-P2-GN5-ET. (6) Dx-P3-GN5-ET. (7) Dx-P3-GN1.8-ET. (8) Gal-P3-ET. (9) Gal-P2-GN5-ET. (10) Gal-P3-GN5-ET. (11) Gal-P3-GN1.8-ET. (12) Dx-P2-ET. (13) Gal-P2-ET. (14) Dx-P2-GN1.8-ET. (15) Gal-P2-GN1.8-ET. (**C**): Analysis by agarose gel electrophoresis of the capacity of siRNA-based vectors with SLN_ED_ to bind the siRNA. (1) 10–300 bp DNA ladder. (2) Naked siRNA. (3) siRNA- GN5. (4) Dx-P3-ED. (5) Dx-P2-GN5-ED. (6) Dx-P3-GN5-ED. (7) Dx-P3-GN1.8-ED. (8) Gal-P3-ED. (9) Gal-P2-GN5-ED. (10) Gal-P3-GN5-ED. (11) Gal-P3-GN1.8-ED. (12) Dx-P2-ED. (13) Gal-P2-ED. (14) Dx-P2-GN1.8-ED. (15) Gal-P2-GN1.8-ED.

Figure 4A shows the agarose gel corresponding to the siRNA binding assay to different components and vectors prepared with SLN_HM_. Migration of free siRNA (lane 2) resulted in an intense band on the gel. The weak band observed in lane 3 shows the capacity of the 5 nm GNs to bind siRNA, although not completely, and confirms the results observed in the TEM studies. The complex formed by siRNA, GNs and P showed a weak band (lane 4) with a lower capacity to migrate, indicative of a higher condensation of the siRNA, and that it was completely bound in the complexes also containing Dx or Gal (lanes 5 and 6). Differences in siRNA binding capacity were observed between the SLN_HM_-based vectors (lanes 7–13) depending on the composition. siRNA was not efficiently bound without GNs (lane 11). When 1.8 nm GNs were used (lane 12), the siRNA migrated slightly through the gel, which indicated that the binding was not as strong as with the GN5 vectors. The siRNA complexed with the commercial reagent DharmaFECT^®^ (lane 13) and was able to migrate, leading to a long band being detected on the gel. 

Figure 4B shows the binding capacity of vectors prepared with SLN_ET_. siRNA was completely bound to the vectors containing 5 nm GNs (lanes 4 to 10). siRNA was also bound to the vectors without GNs, but a weak band corresponding to free siRNA was observed, and siRNA was also detected in the loading wells (lanes 12–13), indicating that siRNA was more exposed in those formulations. When the vectors were prepared with GNs of 1.8 nm, free siRNA was observed with the vectors containing the lowest proportion of P (lanes 14 and 15).

In Figure 4C, the agarose gel electrophoresis corresponding to the ED-based vectors is shown. As it happened for SLN_ET_, siRNA was bound completely to the vectors prepared with either P at the highest proportion or 5 nm GNs (lanes 4–11). Nevertheless, for the vectors prepared without GNs or with 1.8 nm GNs, siRNA was detected in the loading wells (lanes 4, 7, 8 and 11). When siRNA was formulated in vectors with P at the lowest proportion without GNs (lanes 12 and 13) or with 1.8 nm GNs (lanes 14 and 15), it was detected in the loading wells or free on the gel.

### 3.5. Cellular Uptake

The cellular uptake 2 h after the addition of the vectors resulted in a rate of positive IMFE-1 cells around 100% (Figure S4), although the vectors containing SLN_ET_ and Gal showed uptake rates ranging from 80 to 90%. Figure S5 shows the intracellular disposition of Nile-Red-labelled vectors.

### 3.6. Intracellular Disposition of the siRNA and GNs

**Figure 5:**
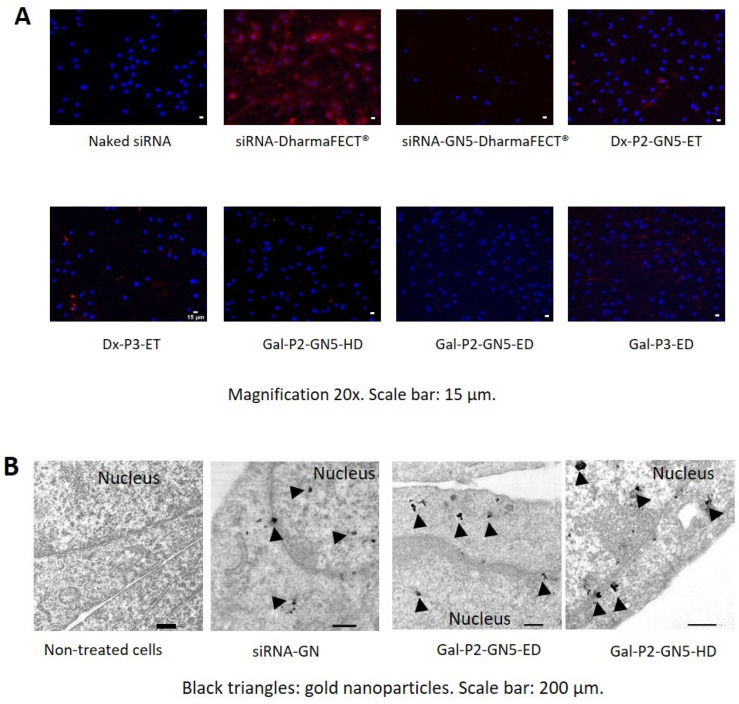
Intracellular disposition of the siRNA and GNs. (**A**): Intracellular disposition of siRNA labelled with Label IT^®^ Cy^®^5 in IMFE-1 cells two hours after transfection studied under fluorescence microscopy. Blue: Nuclei labelled with DAPI. Red: fluorescence signal of nucleic acid labelled with Cy^®^5. (**B**): TEM images of IMFE-1 cells 20 min after been transfected with siRNA vectors containing GNs.

To trigger RNA interference processing, siRNA has to interact with cytosolic RNA interference proteins, such as Ago2, and induce cleavage of target mRNA in a sequence-specific manner. Accordingly, elevated cytosolic location of siRNA is usually a crucial factor for efficacy [39]. Figure 5A shows the intracellular disposition of Label IT^®^ Cy^®^5 siRNA vectors in IMFE-1 cells. No fluorescence was detected with naked siRNA. On the contrary, siRNA appeared extensively distributed in IMFE-1 cells treated with DharmaFECT^®^, a lipid-based commercial transfection reagent. The high degree of siRNA condensation due to the GNs, the P and the cationic lipids may be responsible for the difficulty to detect siRNA in the cells treated with the vectors. In fact, the fluorescence was hardly detected when siRNA was complexed with GNs and then formulated in DharmaFECT^®^, confirming the high capacity of GNs to condense the siRNA.

Figure 5B shows TEM pictures of IMFE-1 cells 20 min after transfection with siRNA-GN5, Gal-P2-GN5-ED and Gal-P2-GN5-HD. In all cases, GNs were located in both the cytoplasm and inside the nucleus. Considering the nucleus as the target location for long-term silencing effect, golden LPNs could represent a promising vector since they also contain P, effective for nuclear localization [40], and 5 nm GNs, which enhance nuclear targeting and ensure their entry by fusion processes [41,42]. siRNA can act by the direct post-transcriptional degradation of the complementary mRNA into the cytoplasm, as well as by transcriptional methylation of homologous DNA sequences [43,44]. In addition, the incorporation to golden LNPs of antisense oligonucleotides (ASOs) could be a promising strategy to produce a prolonged-silencing effect, since they can target RNA either in cytoplasm or in the nucleus (pre-mRNA, mRNA, non-coding RNA, etc.) [45,46].

### 3.7. Silencing Efficacy 

**Figure 6:**
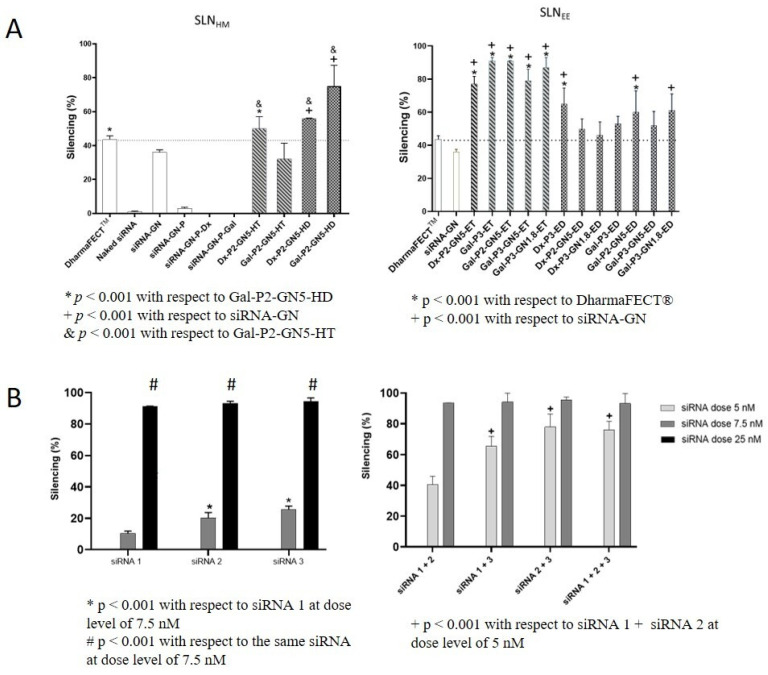
Silencing efficacy of siRNA vectors. (**A**): Gb3S silencing efficacy of the SLN-based vectors with siRNA 1 at 25 nM in IMFE-1 cells 48 h post-transfection measured by RT-qPCR. (**B**): Effect on the Gb3S silencing efficacy of siRNA dose of Gal-P3-GN5-ET containing different siRNA and different siRNA mixtures. Data are expressed as mean ± standard deviation, *n* = 4.

IMFE-1 cells show significantly reduced α-Gal A activity and an accumulation of Gb3 in lysosomes [23]. This cell line provides a useful in vitro model of FD and facilitates systematic studies to explore new therapeutic approaches for this LSD [47]. Figure 6A shows the silencing efficacy of the siRNA-based vectors in IMFE-1 cells at an siRNA dose of 25 nM. As it can be seen, naked siRNA hardly showed the silencing effect (<2%). To be effective, siRNA had to be combined with either GNs, SLNs, or GNs and SLNs. Although siRNA-GNs complexes achieved a 36% silencing rate, GNs lack serum stability and have a very short self-life [48]. Thus, the combination of GNs with other components is a common strategy. Nevertheless, siRNA-P and siRNA-GN-P complexes, with and without Dx or Gal, did not show silencing activity. In contrast, SLN-based vectors silenced Gb3S, although the efficacy varied depending on the composition of the vectors and the preparation method. Regarding SLN_HM_ vectors, Gal-P2-GN5-HD showed the highest silencing effect with a rate of 75%, significantly superior to DharmaFECT^®^ (*p* < 0.001). SLN_ET_-based vectors were the most effective, regardless whether they contained GNs or not, with a silencing rate ranging from 77% to 91%.

Differences observed in transfection are not related to the quantitative uptake of the vectors by IMFE-1 cells. In fact, Gal-ET vectors presented the lowest cellular entry, around 80% (Figure S4), but they produced the highest silencing effect (Figure 6). Therefore, the intracellular behavior of Gal-ET vectors seems to be more effective to silence Gb3S. The efficacy of the vectors depends on the balance between the protection degree of the nucleic acid in the formulation and the ability to be released into the cytoplasm [22]. Nanovectors may use different internalization mechanisms simultaneously to enter into the cell, which condition their fate. In fact, one of the limiting steps for siRNA delivery to date is to prevent the endocytosed nanoparticles being routed toward endolysosomes [49,50]. Permanent cationic lipids, such as DOTAP, enhance the cellular uptake, while conditionally ionized cationic lipids with pKa values of 6–7, such as DODAP, improve endosomal escape and other intracellular delivery routes [51,52]. The inclusion of DODAP in SLN-based systems for siRNA delivery has not been evaluated until now, although nanomedicines with a combination of both permanent and pH-sensitive cationic lipids are expected to result in enhanced gene silencing [53,54]. SLN_HM_ and Gal vectors showed improved silencing efficacy when DODAP was included in the formulation. Moreover, different studies have reported the influence of DODAP on the biodistribution and the in vivo efficacy of nucleic acids formulated in LNP [55,56]. 

Recently, machine learning methods have suggested that the sequence and thermodynamic parameters of siRNA are strongly associated with the effectiveness of gene silencing [57]. Therefore, by using Gal-P3-GN5-HT, we tested three different siRNA sequences targeted to Gb3S and their combinations. Figure 6B shows the silencing rate for the three siRNA against Gb3S at different doses and for different combinations of siRNA. Silencing activity was dose-dependent and, regardless of the siRNA, gene suppression > 85% was achieved at the level dose of 25 nM. However, when a mixture of siRNAs was evaluated, a final dose of 7.5 nM was enough to achieve a silencing effect of 90%, allowing a three-fold reduction in the required dose. Although the combination of the three siRNA provided high silencing activity, it did not improve over the combined use of the two that were most effective (siRNA 2 + siRNA 3).

### 3.8. Gb3S Enzyme Expression

**Figure 7:**
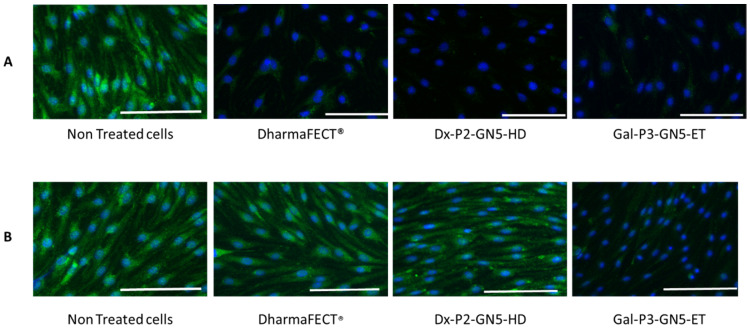
Fluorescence microscopy images of the immunocytochemistry assay of Gb3S in IMFE-1 cells transfected with the siRNA-based vectors. (**A**) Study 7 days after transfection. (**B**) Study 15 days after transfection. Blue: Nuclei labeled with DAPI. Green: Fluorescence signal of Gb3S. Magnification: 20×. Scale bar: 15 µm.

The efficacy of the nanocarriers to reduce the Gb3S enzyme expression in IMFE-1 cells was evaluated by immunocytochemistry. Figure 7 shows fluorescence microscopy images corresponding to non-treated cells, and cells treated with DharmaFECT^®^, Dx-P2-GN5-HD, and Gal-P3-GN5-ET, 7 (Figure 7A) at 15 days (Figure 7B) after transfection (additional images are showed in Figures S6 and S7). While green fluorescence corresponding to Gb3S was very abundant in non-treated cells (it was detected in every cell), it was hardly detected in the cells treated with the nanovectors at day 7, and a small amount was detected in the cells treated with DharmaFECT^®^. Fifteen days after transfection, the reduction in Gb3S expression was maintained in cells treated with Gal-P2-GN5-ET vectors. These immunocytochemistry studies showed differences in long-term efficacy depending on the formulation and confirmed the highest silencing activity of the vectors containing SLN_EE_ and GNs. 

Specific SRT for FD based on the inhibition of Gb3S has not been widely studied, and studies about Gb3S silencing are scarce [58,59]. To the best of our knowledge, only one study, published very recently, has reported the development of a delivery system with siRNA targeted to Gb3S [60]. The system, based on the combination of poly-histidine and LNPs, silenced the Gb3S mRNA expression in A549 cells (adenocarcinomic human alveolar basal epithelial cells). In the present study, we confirm, for the first time, not only the downregulation of Gb3S mRNA expression but also the reduction in Gb3S enzymes with an siRNA-targeted Gb3s strategy using LNP-based vectors, with high efficacy in a cell model of the disease. The novel siRNA delivery system represents an interesting SRT strategy to treat other LSDs, since the vectors could be designed to target the specific enzymes implied in the synthesis of the corresponding accumulation product for each LSD.

### 3.9. Cell Viability

Cell viability was around 100% when treated with the vectors at an siRNA dose of 25 nM (Figure S8), and cell viability at 50 nM dose was higher than 80. At the higher dose level, DharmaFECT^®^ provided a cell viability of 71%.

### 3.10. Interaction with Erythrocytes: Hemolysis and Hemagglutination

**Figure 8:**
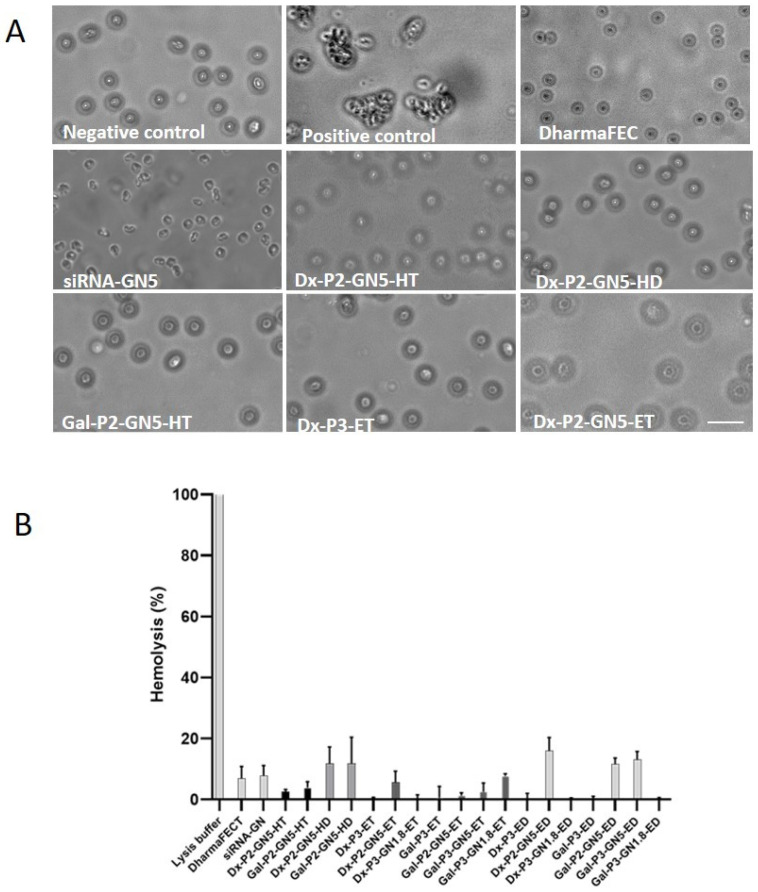
(**A**) Images of untreated red cells (negative control) and treated with the siRNA vectors corresponding to the hemagglutination study. Positive control: Poly-*L*-Lysine. Scale bar: 50 µm. (**B**) Hemolytic effect of the siRNA-based vectors. Lysis buffer represents 100% hemolysis sample. Results are shown as mean ± standard deviation; *n* = 3.

A possible drawback for in vivo application of cationic delivery systems is the risk of interaction with erythrocytes and other blood components, such as serum proteins [61]. A very slight agglutination was detected after the treatment of the erythrocytes with the siRNA-GNs complex, whereas the formulations containing SLNs did not induce agglutination of the red cells (Figure 8A). The hemolytic activity of the siRNA vectors (Figure 8B) was lower than 15% for all nanovectors, although some differences depending on the composition were detected. 

Golden LNPs showed good hemocompatibility. However, contradictory studies have been published regarding GNs toxicity, and more studies are required [62,63]. Different parameters of GNs are related to systemic toxicity, such as size, shape and surface chemistry [63,64]. GNs smaller than 10 nm have demonstrated low systemic toxicity and fast kidney clearance [65,66]. The surface chemistry characteristics and the size of GNs are also related to cellular toxicity of GNs, although studies are also controversial [67,68,69].

In spite of the promising results, our study presents some limitations. On the one hand, we have shown the stability of the vectors for one month, but a longer follow-up study would confirm their long-term stability. On the other hand, we have evaluated the silencing efficacy of the nanosystems using RT-qPCR, which measures the mRNA expression, and using immunocytochemistry to detect the reduction in the enzyme in the cytoplasm of the cells; however, the reduction in Gb3 accumulation in IMFE-1 cells would yield valuable insights into the potential of this method for future investigations. Finally, although the vectors demonstrated capacity to downregulate the Gb3S in vitro in a FD cell model, the lack of in vitro–in vivo correlation in the gene therapy field is well known. Therefore, future in vivo studies in animal models of the disease would help to confirm the potential of our formulations to treat FD.

## 4. Conclusions

In this study, a novel siRNA nanomedicine based on golden LNPs with a high Gb3S silencing capacity, and therefore, with application in FD, has been developed. The composition, the order of addition of the components and the method of preparation of SLNs resulted in key parameters for efficacy, with golden LNPs prepared from SLN_EE_ with the cationic lipid DOTAP being the most effective. The presence of GNs within the nanocarrier favored the interaction with the siRNA and thus improved the payload capacity and stability of the golden LNPs, resulting in a great advantage in terms of efficacy, as well as physical properties and versatility of the final nanosystem. The treatment of the IMFE-1 cells with a single dose of the siRNA nanomedicine led to a significant reduction in the expression of Gb3S enzyme, which was maintained for up to two weeks. These results show the potential of the siRNA–golden LNPs targeted to Gb3S for the treatment of FD by specific SRT.

## Figures and Tables

**Table 1:** Composition and physicochemical characteristics of the SLNs.

Type of SLN	Preparation Method	Lipid (%)		Tween 80 (%)	Size (d.nm)	PDI	ζ-Potential (mV)
		DOTAP	DODAP				
SLNHT	HM	0.4	-	0.1	90.0 ± 0.8	0.27 ± 0.01	+67.2 ± 0.7
SLNHD	HM	0.2	0.2	0.1	119.3 ± 0.6	0.23 ± 0.01	+71.3 ± 0.9
SLNET	EE	0.4	-	0.1	181.2 ± 5.5	0.26 ± 0.03	+58.4 ± 1.6
SLNED	EE	0.2	0.2	0.1	218.8 ± 1.2	0.26 ± 0.05	+52.2 ± 1.6

HM: Hot melt, EE: emulsification/evaporation, SLN: solid lipid nanoparticle, SLNHT: SLN prepared by HM with DOTAP, SLNHD: SLN prepared by HM with DOTAP/DODAP, SLNET: SLN prepared by EE with DOTAP, SLNED: SLN prepared by EE with DOTAP/DODAP. Data are expressed as mean ± standard deviation; n = 3. Significant differences (*p* < 0.001) in size and zeta potential were observed among all formulations.

## Data Availability

The data presented in this study are available upon request from the corresponding author.

## Supplementary Materials

The following supporting information can be downloaded at: https://www.mdpi.com/article/10.3390/pharmaceutics15071936/s1, Table S1: Composition of siRNA-based vectors; Table S2: Physical characterization of SLNs-based vectors; Figure S1: Preparation of siRNA-based vectors; Figure S2: Size, ζ-Potential and polydispersity index of siRNA-based vectors formulated with different SLNs at time zero, ten, twenty and thirty days; Figure S3: Cellular uptake of vectors using Nile-Red-labelled SLNs in IMFE-1 cells 2 h after transfection measured by flow cytometry; Figure S4: Cellular uptake of vectors using Nile-Red-labelled SLNs in IMFE-1 cells 2 h after transfection obtained by fluorescent microscopy; Figure S5: Study of the silencing capacity of different siRNA at 15, 25 and 50 nM; Figure S6: Immunocytochemistry study of Gb3S expression in IMFE-1 cells 7 days after treatment with different siRNA-based vectors; Figure S7: Immunocytochemistry study of Gb3S expression in IMFE-1 cells 15 days after treatment with different siRNA-based vectors; Figure S8: Viability in IMFE-1 cells two hours after the addition of siRNA-based vectors analyzed by MTT assay.
