# Supplementary material for: Novel Golden Lipid Nanoparticles with Small Interference Ribonucleic Acid for Substrate Reduction Therapy in Fabry Disease
Source: Pharmaceutics. 2023 Jul 12;15(7):1936. doi: 10.3390/pharmaceutics15071936 (PMC10385692; doi:10.3390/pharmaceutics15071936)
Supplement: Supplementary file 1 [file pharmaceutics-15-01936-s001.zip › pharmaceutics-2467004-supplementary.pdf]

Supplementary material

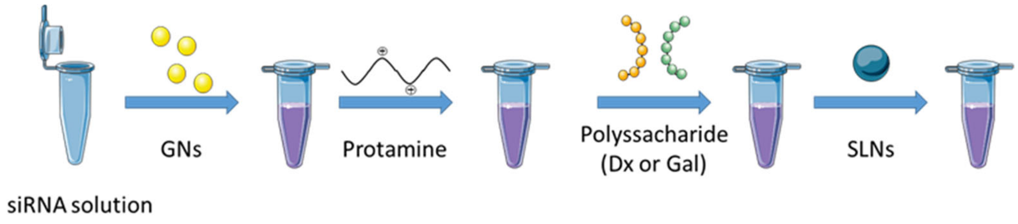

Figure S1. Preparation of siRNA-based vectors.

Table S1. Composition of siRNA-based vectors.

Vector	Type of SLN	Dx:P:siRNA (w:w:w ratio)	Gal:P:siRNA (w:w:w ratio)	Size of GN (nm)
DX-P2-GN5-HT	HT	1:2:1		5
DX-P3-GN5-HT	HT	1:3:1		5
Gal-P2-GN5-HT	HT		0.1:2:1	5
Gal-P3-GN5-HT	HT		0.1:3:1	5
DX-P2-GN5-HD	HD	1:2:1		5
DX-P3-GN5-HD	HD	1:3:1		5
Gal-P2-GN5-HD	HD		0.1:2:1	5
Gal-P3-GN5-HD	HD		0.1:3:1	5
DX-P3-ET	ET	1:3:1		-
DX-P2-GN5-ET	ET	1:2:1		5
DX-P3-GN5-ET	ET	1:3:1		5
DX-P2-GN1.8-ET	ET	1:2:1		1.8
DX-P3-GN1.8-ET	ET	1:3:1	0.1:3:1	1.8
3Gal-P3-ET	ET		0.1:3:1	-
Gal-P2-GN5-ET	ET		0.1:2:1	5
Gal-P3-GN5-ET	ET		0.1:3:1	5
Gal-P2-GN1.8-ET	ET		0.1:2:1	1.8
Gal-P3-GN1.8-ET	ET		0.1:3:1	1.8
DX-P3-ED	ED	1:3:1		-
DX-P2-GN5-ED	ED	1:2:1		5
DX-P3-GN5-ED	ED	1:3:1		5
DX-P2-GN1.8-ED	ED	1:2:1		1.8
DX-P3-GN1.8-ED	ED	1:3:1		1.8
Gal-P3-ED	ED		0.1:3:1	-
Gal-P2-GN5-ED	ED		0.1:2:1	5
Gal-P3-GN5-ED	ED		0.1:3:1	5
Gal-P2-GN1.8-ED	ED		0.1:2:1	1.8

Gal-P3-GN1.8-ED	ED		0.1:3:1	1.8

HT: HT-based vectors. HD: HD-based vectors. ET: ET-based vectors. ED: ED-based vectors. DX: dextran. Gal: galactomannan. P: protamine. GN5: gold nanoparticles of 5 nm diameter. GN1.8: gold nanoparticles of 1.8 nm diameter. PDI: polidespersity index. SLNs were added in a weight ratio of 5 referred to siRNA. GN of 5 nm were added in a volume of 5 µL while 1 µL of 1.8 nm GN were used. Data are expressed as mean ± standard deviation; *n*=3.

Table S2. Physical characterization of SLNs-based vectors.

SLN-based vector	Vector	Size Z-average (d.nm)	PDI	ζ-Potential (mV)
HT	DX-P2-GN5-HT	173.3 ± 55.2	0.35 ± 0.08	38.8 ± 8.3
	DX-P3-GN5-HT	237.2 ± 14.8	0.44 ± 0.05	30.9 ± 5.2
	Gal-P2-GN5-HT	278.0 ± 85.0	0.40 ± 0.09	31.7 ± 10.5
	Gal-P3-GN5-HT	314.3 ± 28.9	0.40 ± 0.08	30.0 ± 4.0
HD	DX-P2-GN5-HD	326.8 ± 8.1	0.39 ± 0.04	31.9 ± 8.8
	DX-P3-GN5-HD	294.3 ± 12.8	0.39 ± 0.01	35.9 ± 6.3
	Gal-P2-GN5-HD	273.0 ± 24.6	0.46 ± 0.06	31.7 ± 7.3
	Gal-P3-GN5-HD	270.9 ± 9.9	0.37 ± 0.02	35.4 ± 8.2
ET	DX-P3-ET	280.4 ± 7.3	0.36 ± 0.02	31.2 ± 7.9
	DX-P2-GN5-ET	406.2 ± 21.7	0.39 ± 0.01	31.7 ± 5.7
	DX-P3-GN5-ET	263.3 ± 26.6	0.38 ± 0.01	31.0 ± 6.6
	DX-P2-GN1.8-ET	195.2 ± 6.5	0.35 ± 0.05	30.2 ± 7.1
	DX-P3-GN1.8-ET	284.3 ± 18.3	0.36 ± 0.01	32.5 ± 7.6
	Gal-P3-ET	280.6 ± 7.5	0.36 ± 0.02	40.8 ± 8.0
	Gal-P2-GN5-ET	381.9 ± 43.9	0.45 ± 0.03	38.3 ± 6.2
	Gal-P3-GN5-ET	276.5 ± 13.7	0.37 ± 0.04	35.5 ± 6.8
	Gal-P2-GN1.8-ET	219.7 ± 9.7	0.34 ± 0.09	36.8 ± 8.5
	Gal-P3-GN1.8-ET	274.1 ± 8.6	0.39 ± 0.04	32.7 ± 7.8
ED	DX-P3-ED	337.5 ± 15.3	0.33 ± 0.03	33.8 ± 7.7
	DX-P2-GN5-ED	336.8 ± 69.7	0.40 ± 0.07	28.5 ± 7.9
	DX-P3-GN5-ED	336.5 ± 7.9	0.31 ± 0.04	35.5 ± 9.3
	DX-P2-GN1.8-ED	311.7 ± 22.2	0.44 ± 0.03	34.0 ± 7.6
	DX-P3-GN1.8-ED	304.1 ± 21.6	0.33 ± 0.05	32.6 ± 8.4
	Gal-P3-ED	300.3 ± 7.9	0.34 ±0.02	32.7 ± 10.1
	Gal-P2-GN5-ED	221.5 ± 21.5	0.37 ±0.09	33.9 ± 9.5
	Gal-P3-GN5-ED	240.9 ± 10.0	0.40 ± 0.06	35.4 ± 8.1
	Gal-P2-GN1.8-ED	301.1 ± 8.6	0.40 ± 0.05	34.9 ± 10.0
	Gal-P3-GN1.8-ED	278.9 ± 13.4	0.41 ± 0.05	36.5 ± 6.4

HT: HT-based vectors. HD: HD-based vectors. ET: ET-based vectors. ED: ED-based vectors. DX: dextran. Gal: galactomannan. P: protamine. GN5: gold nanoparticles of 5 nm diameter. GN1.8: gold nanoparticles of 1.8 nm diameter. PDI: polidispersity index. Data are expressed as mean ± standard deviation; *n*=3.

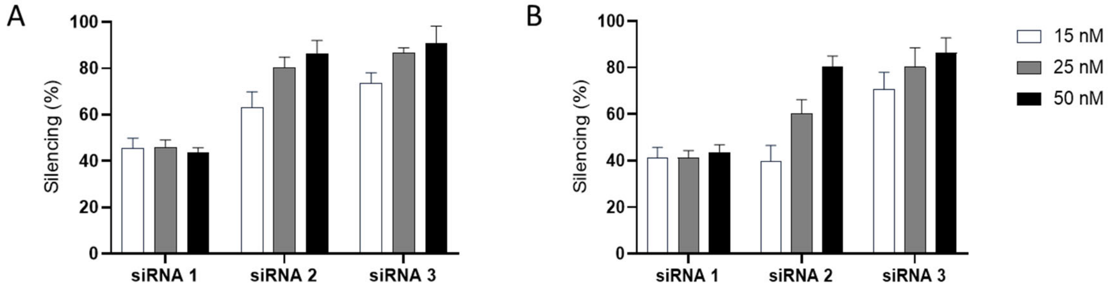

Figure S2. Study of the silencing capacity of different siRNA at 15, 25 and 50 nM. (**A**) Silencing effect of siRNAs 1, 2 and 3 formulated in Dx-P2-GN5-HT. (**B**) Silencing effect of siRNAs 1, 2 and 3 formulated with DharmaFECT®. Data are expressed as mean ± standard deviation; n=3.

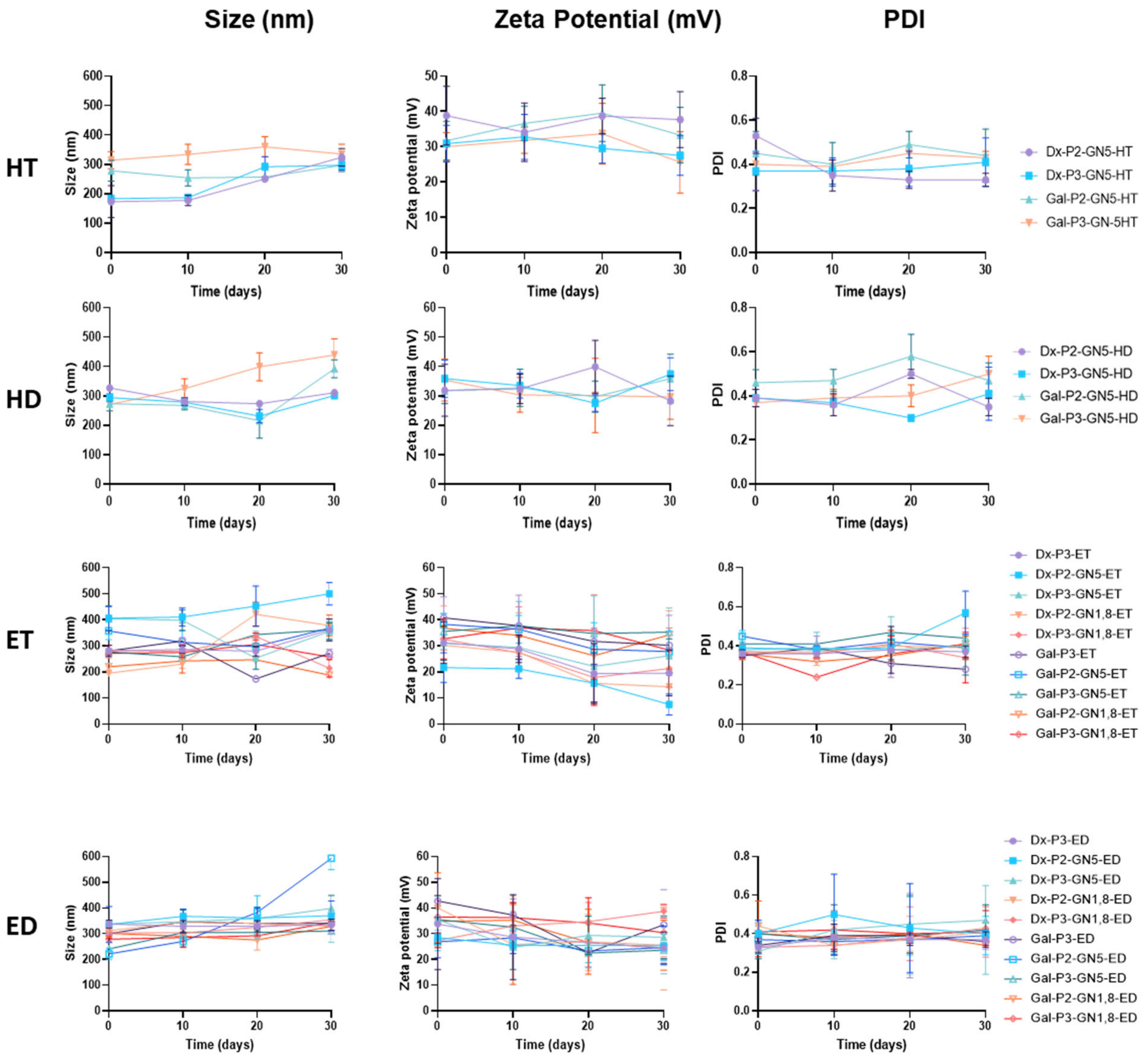

Figure S3. Size, ζ-Potential and polydispersity index of siRNA-based vectors formulated with different SLNs at time zero, ten, twenty and thirty days. (**HT**) HT- Vectors. (**HD**) HD-Vectors. (**ET**) ET-Vectors. (**ED**) ED-Vectors. Data are expressed as mean ± standard deviation; n=3.

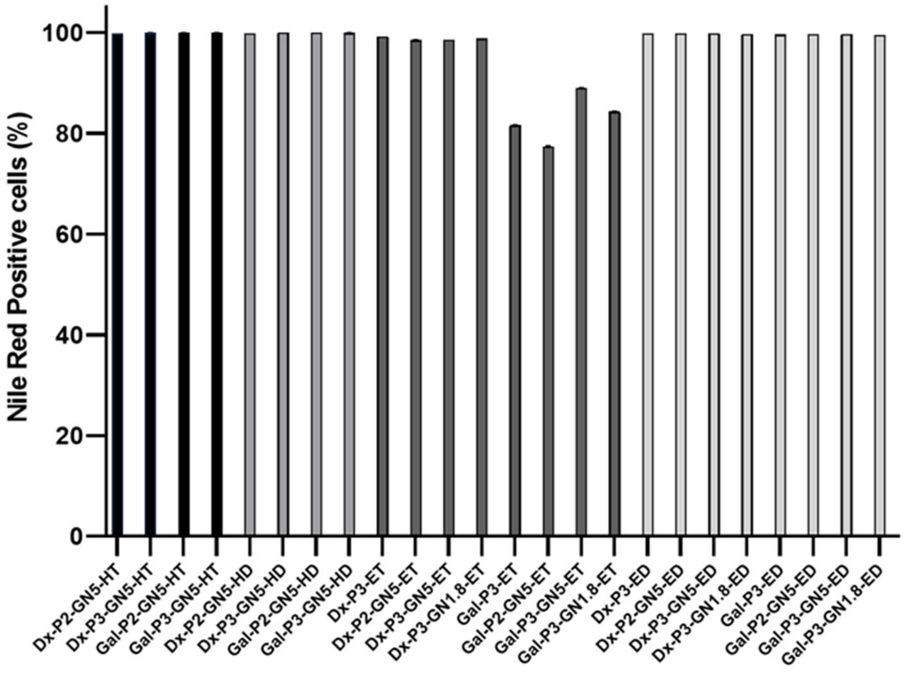

Figure S4. Cellular uptake of vectors using Nile-Red labelled SLNs in IMFE-1 cells 2 hours after transfection measured by flow cytometry. Data are expressed as mean ± standard deviation; n=3.

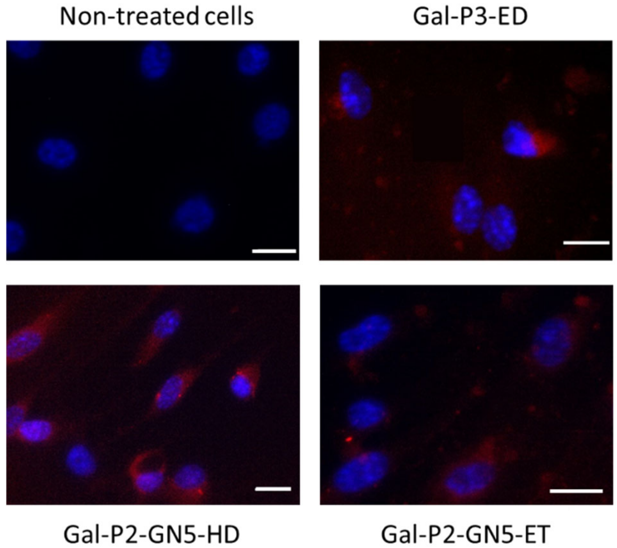

Figure S5. Cellular uptake of vectors using Nile-Red labelled SLNs in IMFE-1 cells 2 hours after transfection obtained by fluorescent microscopy. Scale bar = 15 μm.

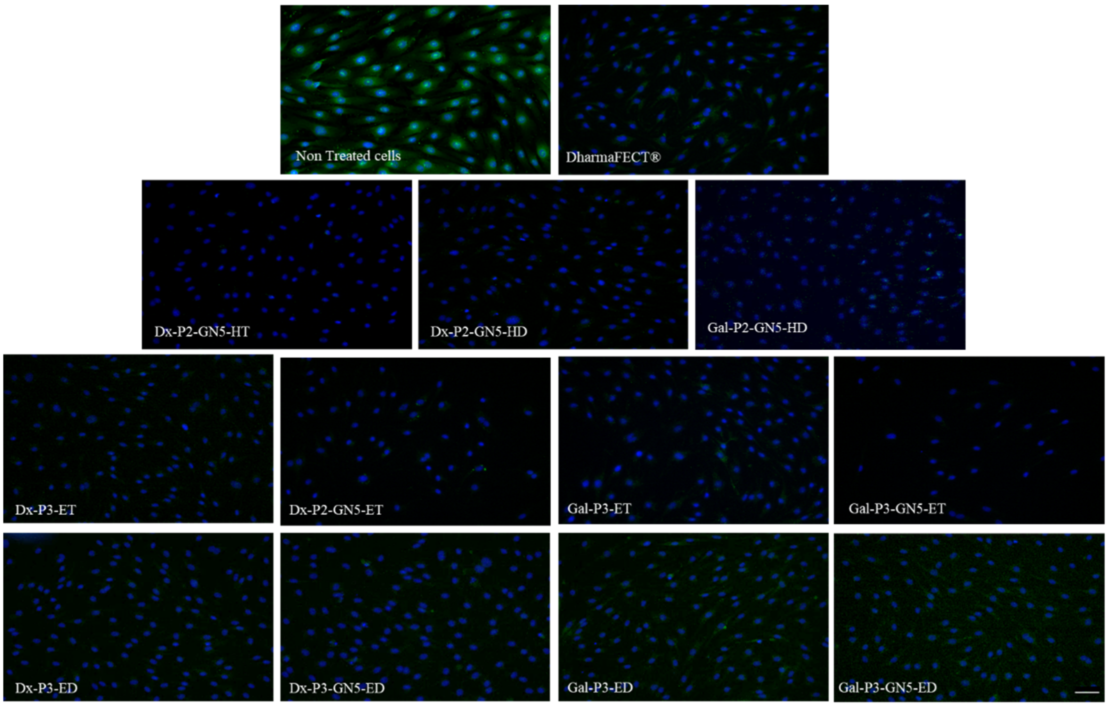

Figure S6. Immunocytochemistry study of Gb3S expression in IMFE-1 cells 7 days after treatment with different siRNA-based vectors. Scale bar: 100 µm.

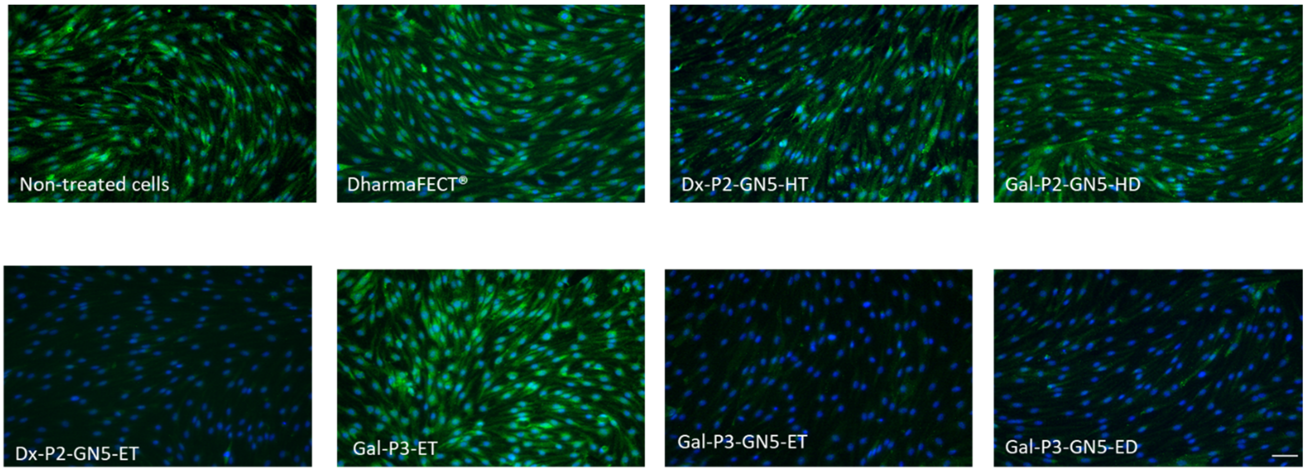

Figure S7. Immunocytochemistry study of Gb3S expression in IMFE-1 cells 15 days after treatment with different siRNA-based vectors. Scale bar: 100 µm.

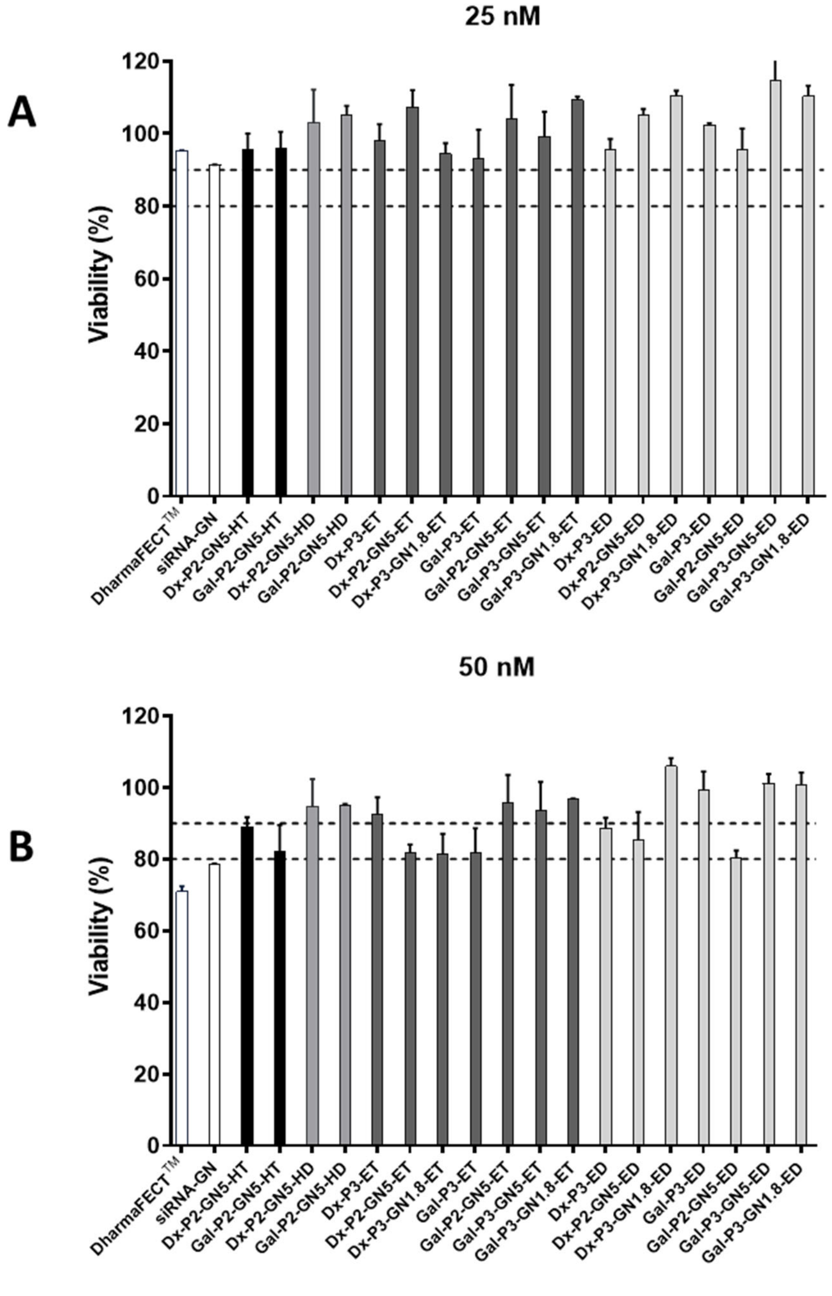

Figure S8. Viability in IMFE-1 cells two hours after the addition of siRNA-based vectors analyzed by MTT assay. Data are expressed as mean ± standard deviation; n=3. (**A**) Viability in IMFE-1 after the addition of vectors a dose 25 nM. (**B**) Viability in IMFE-1 cells after the addition of vectors of vector at a dose 50 nM. Lines represent 80% and 90%.
